# PPAR**γ** Agonist Rosiglitazone Suppresses Renal mPGES-1/PGE2 Pathway in db/db Mice

**DOI:** 10.1155/2013/612971

**Published:** 2013-12-30

**Authors:** Ying Sun, Zhanjun Jia, Gang Liu, Li Zhou, Mi Liu, Baoxue Yang, Tianxin Yang

**Affiliations:** ^1^Department of Pharmacology, School of Basic Medical Sciences, Peking University, 38 Xueyuan Road, Haidian District, Beijing 100191, China; ^2^Department of Internal Medicine, University of Utah and Veterans Affairs Medical Center, Salt Lake City, UT, USA; ^3^Division of Nephrology and Hypertension, University of Utah and Veterans Affairs Medical Center, 30N 1900E, RM 4C224, Salt Lake City, UT 84132, USA

## Abstract

Evidence had shown the detrimental effect of prostaglandin (PG) E2 in diabetic nephropathy (DN) of STZ-induced type-1 diabetes but its role in the development of DN of type-2 diabetes remains uncertain. The present study was undertaken to investigate the regulation of PGE2 synthetic pathway and the interaction between peroxisome proliferator-activated receptor (PPAR)**γ** and PGE2 synthesis in the kidneys of db/db mice. Strikingly, urinary PGE2 was remarkably elevated in db/db mice paralleled with the increased protein expressions of COX-2 and mPGES-1. In contrast, the protein expressions of COX-1, mPGES-2, cPGES, and 15-hydroxyprostaglandin dehydrogenase (15-PGDH) were not altered. Following 1-week rosiglitazone (Rosi) therapy, urinary PGE2, but not other prostanoids, was reduced by 57% in parallel with significant reduction of mPGES-1 protein and EP4 mRNA expressions. By immunohistochemistry, mPGES-1 was significantly induced in the glomeruli of db/db mice, which was almost entirely abolished by Rosi. In line with the reduction of glomerular mPGES-1, the glomerular injury score showed a tendency of improvement after 1 week of Rosi therapy. Collectively, the present study demonstrated an inhibitory effect of PPAR**γ** activation on renal mPGES-1/PGE2/EP4 pathway in type-2 diabetes and suggested that mPGES-1 may potentially serve as a therapeutic target for treating type-2 diabetes-associated DN.

## 1. Introduction

The abnormality of renal prostaglandins in diabetic kidney was thought to be an important factor in mediating the glomerular injury, tubular interstitial fibrosis, and fluid imbalance [1–4]. Among five prostanoids of PGE2, PGD2, PGI2, PGF2*α*, and thromboxane (TX) A2, PGI2 and TXA2 are important players in regulation of renal hemodynamics [5–7]; PGE2 serves as an important regulator of glomerular integrity, SS hemodynamics, and tubular fluid reabsorption [8–10], while the PGD2 and PGF2*α* in kidney are less studied and their functions are poorly understood. 

The renal PGE2 receptor (EP1-EP4) expression profile is altered in type-1 diabetic mice [11]. Makino et al. demonstrated that EP1-selective antagonist prevented the progression of nephropathy in streptozotocin (STZ) diabetic rats [12]. Most recently, Mohamed et al. reported that EP4 agonist exacerbated kidney injury in STZ-induced type-1 diabetes in mice [13]. These reports strongly suggested a detrimental role of PGE2 in kidney of STZ-induced type-1 diabetes via EP1 and/or EP4 receptors. However, no prior studies examine the role of PGE synthase in the kidney injury of type-2 diabetes. In clinic, type-2 diabetes is more common than type-1 diabetes and the kidney injury of type-2 diabetes possesses more complex pathogenic mechanism than that in type-1 diabetes. For animal studies of type-2 diabetes, the leptin receptor mutant db/db mice are widely used [14, 15]. Our recent publication demonstrated the induction of renal COX-2 and PGE2 in db/db mice [16], but the entire renal profile of prostaglandins, particularly the PGE2-related synthases, remains uncertain in this mouse model.

PPAR*γ* agonists including rosiglitazone and piglitazone are very effective agents in treatment of type-2 diabetes. Besides their insulin sensitizing effect, PPAR*γ* agonists also protect the organs from various injuries via anti-inflammation, antioxidative stress, and antiapoptosis [17, 18]. Although numerous lines of evidence demonstrated the beneficial effect of PPAR*γ* activation on the diabetic kidney injury [19–22], the role of PPAR*γ* in the regulation of renal prostaglandins in diabetic kidney is still unknown. In the present study, we investigated (1) the regulation of renal COX-2/mPGES-1/PGE2/EP pathway in obese-diabetic db/db mice and (2) the effect of PPAR*γ* agonist Rosi on such a regulation.

## 2. Methods

### 2.1. Animals and Treatments

Obese-diabetic Lepr^db/db^ (db/db, B6.BKS(D)-leprdb/J), Lepr^db/m^ (db/m, lean control), and C57/BL6 male mice were purchased from the Jacson Laboratories (Bar Harbor, ME). In all studies, 7- to 8-month-old male mice were used. The mice were divided into three groups: db/m control (lean, *n* = 4), db/db without Rosi (db/db, *n* = 5), and db/db with Rosi (db/db + Rosi, *n* = 5). The Rosi was administered to db/db mice in jelly diet (80 mg/kg food) for 1 week as described previously [23]. The db/m control and db/db control mice were given the same jelly diet without Rosi. The Rosi maleate (Cat no. BRL49653C) was provided by GlaxoSmithKline (Harlow, UK). All mice were maintained under a 12:12-h light-dark cycle (lights on at 6:00 a.m. and lights off at 6:00 p.m.). All protocols employing mice were conducted in accordance with the principles and guidance of the University of Utah Institutional Animal Care and Use Committee.

### 2.2. Immunoblotting

The whole kidney was lysed and protein concentration was determined by Coomassie reagent. Protein (60 *μ*g) from whole kidney lysates was denatured in boiling water for 10 min, separated by SDS-polyacrylamide gel electrophoresis, and transferred onto nitrocellulose membranes. The blots were blocked overnight with 5% nonfat dry milk in Tris-buffered saline (TBS), followed by incubation for 1 h with rabbit anti-COX-1 (Cayman Chemicals, Cat no. 160108), anti-COX-2 (Cayman Chemicals, Cat no. 160106), anti-mPGES-1 (Cayman Chemicals, Cat no. 160140), anti-mPGES-2 (Cayman Chemicals, Cat no. 160145), anti-cPGES (Cayman Chemicals, Cat no. 160150), anti-15-PGDH (Cayman Chemicals, Cat no. 160615), anti-PPAR*γ* (Santa Cruz, CAT no. SC-7273), and anti *β*-actin (Sigma Aldrich, Cat no. A1978) at a dilution of 1 : 1000. After being washed with TBS, blots were incubated with a goat anti-horseradish peroxidase-conjugated secondary antibody (1 : 1000 dilution) and visualized with ECL kits (Amersham, Piscataway, NJ, USA).

### 2.3. Immunohistochemistry and Histology

Kidneys from the db/m and db/db mice were fixed with 4% paraformaldehyde and embedded in paraffin. Kidney sections of 4 *μ*m thickness were prepared for the staining. The immunohistochemistry of mPGES-1 was performed using an EnVison TM Mini Kit (Doko, Carpinteria, CA) according to the manufacturer's instruction. The mPGES-1 antibody was purchased from Cayman Chemical (Cat no. 160140) and was applied to the immunohistochemical staining by 1 : 200 dilutions. mPGES-1 specific blocking peptide (Cayman Chemicals, Cat no. 360140) was used to test the specificity of the mPGES-1 antibody signal.

The histology was evaluated by Periodic Acid Schiff (PAS) staining. Semiquantitative scoring of glomerular sclerosis in PAS stained slides was performed using a five-grade method described previously [16, 24]: 0, normal glomerulus; 1, sclerosis <25%; 2, sclerosis between 25 and 50%; 3, sclerosis between 50 and 75%; and 4, sclerosis >75% of glomerular surface. Semiquantitative mesangial area was measured by using computer software Image J (NIH, Bethesda, MD).

### 2.4. qRT-PCR

Total RNA isolation and reverse transcription were performed as previously described [25]. Oligonucleotides were designed using Primer3 software (available at http://frodo.wi.mit.edu/primer3/). The sequences of primers are as follows: EP1 5′-cagtgaagtgcgggtggag-3′(sense) and 5′-tattggggagcctgggtgt-3′(anti-sense) NM_013641; EP4 5′-cttactcatcgccacctct-3′(sense) and 5′-tggggttcacagaagcaatc-3′(anti-sense) NM_008965; GAPDH 5′-gtcttcactaccatggagaagg-3′(sense) and 5′-tcatggatgaccttggccag-3′(anti-sense) M32599. Amplification was performed using the SYBR Green Master Mix (Applied Biosystems, Warrington, UK) and the Prism 7500 Real-Time PCR Detection System (Applied Biosystems, Foster City, CA, USA). Cycling conditions were 95°C for 10 min, followed by 40 repeats of 95°C for 15 s, and 60°C for 1 min. The melting curve was examined and the results were analyzed by delta-delta CT method. GAPDH was used as a reference gene.

### 2.5. Enzyme Immunoassay

Urine samples were centrifuged for 5 minutes at 10000 rpm. Concentrations of PGE2 (Cayman Chemicals, Cat no. 514010), PGD2 (Cayman Chemicals, Cat no. 512031), 6-keto-PGF1*α* (Cayman Chemicals, Cat no. 515211), and TXB2 (Cayman Chemicals, Cat no. 519031) were determined by enzyme immunoassay according to manufacturer's instructions. For the PGE2 assay, the sensitivity or minimal detection limit was 7.8 pg/mL; intra-assay CV was 4.2% and interassay CV 12.4%. For the PGD2 assay, the sensitivity or minimal detection limit was 19.5 pg/mL; intraassay CV was 11% and interassay CV 6.1%. For the TXB2 assay, the sensitivity or minimal detection limit was 7.8 pg/mL; intra-assay CV was 16.3% and interassay CV 26.6%. For the 6-keto PGF1*α* assay, the sensitivity or minimal detection limit was 1.6 pg/mL; intra-assay CV was 6.7% and interassay CV 18.1%.

### 2.6. Statistical Analysis

All values are presented as mean ± SE. Statistical analysis was performed using ANOVA. Differences were considered to be significant when *P* < 0.05.

## 3. Results

### 3.1. Effect of Rosi Treatment on Urinary Prostanoids Output in db/db Mice

**Figure 1:**
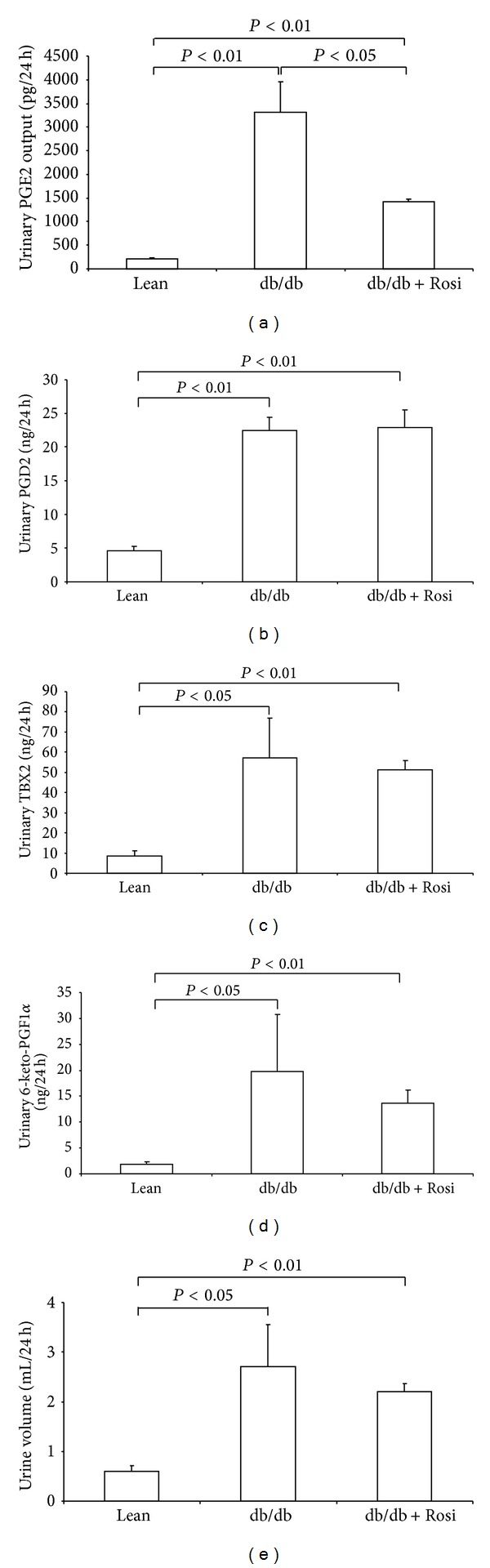
Effect of Rosi treatment on the urinary output of prostanoids and urine volume in db/db mice. (a) Urinary PGE2 output. (b) Urinary PGD2 output. (c) Urinary TXB2 output. (d) Urinary 6-keto-PGF1*α* output. (e) Urinary volume. *N* = 4-5 in each group. Data are mean ± SE.

The urinary PGE2 output in db/db mice was markedly elevated compared with the age matched lean mice (Figure 1(a)), and such an increase was significantly blunted by Rosi treatment (Figure 1(a)). In addition, the baseline blood glucose level in this obese-diabetic db/db mouse strain was higher than lean controls (db/db: 160.1 ± 18.7 mg/dL versus lean: 100.0 ± 7.3 mg/dL, *P* < 0.05). One-week Rosi treatment entirely normalized the blood glucose (104.6 ± 8.1 mg/dL). The urinary PGD2 (Figure 1(b)), TXB2 (stable metabolite of TXA2) (Figure 1(c)), and 6-keto-PGF1*α* (stable metabolite of PGI2) (Figure 1(d)) were all remarkably increased in the db/db mice, which was unaffected by Rosi administration. The urine volume was not affected following 1-week Rosi therapy (Figure 1(e)).

### 3.2. Effect of Rosi on the Protein Expressions of Prostaglandin E Synthases in db/db Mice

**Figure 2:**
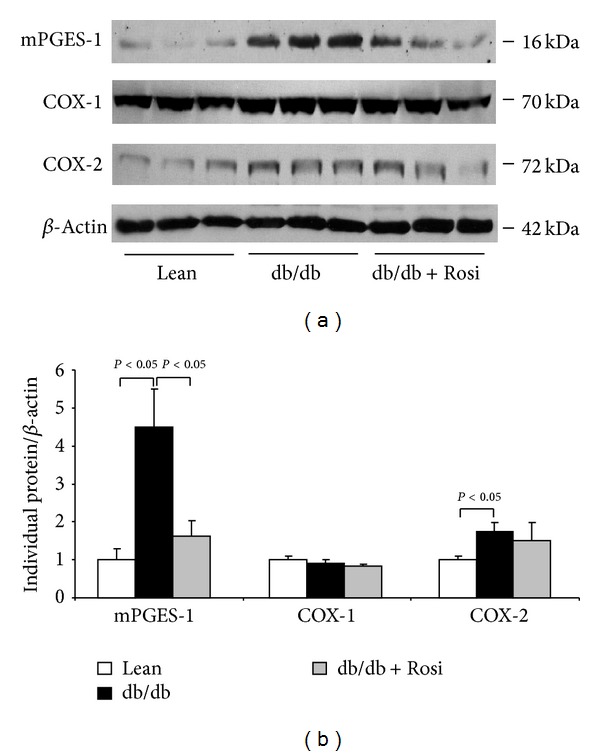
Effect of Rosi treatment on the protein expressions of mPGES-1, COX-1, and COX-2 in the kidneys of db/db mice. (a) Western blots of mPGES-1, COX-1, COX-2, and *β*-actin. (b) Densitometry of western blots. *N* = 4-5 in each group. Data are mean ± SE.

**Figure 3:**
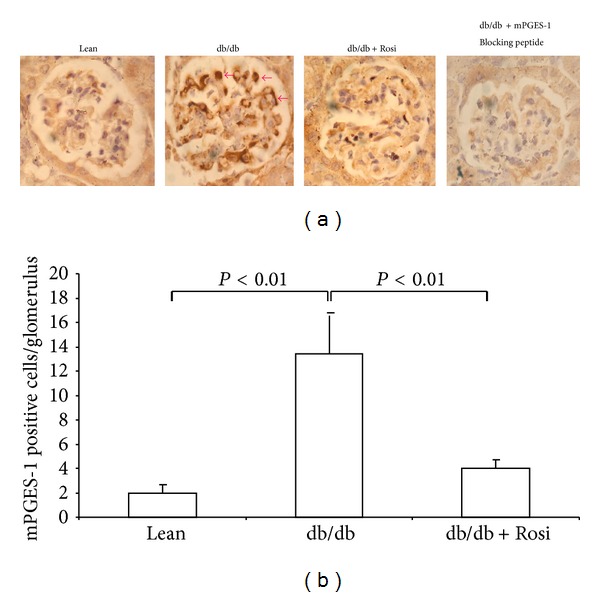
Effect of Rosi treatment on glomerular mPGES-1 expression in db/db mice. (a) Immunohistochemstry of mPGES-1. (b) Quantification of mPGES-1 positive cells in the glomeruli. *N* = 4-5 in each group. Data are mean ± SE.

**Figure 4:**
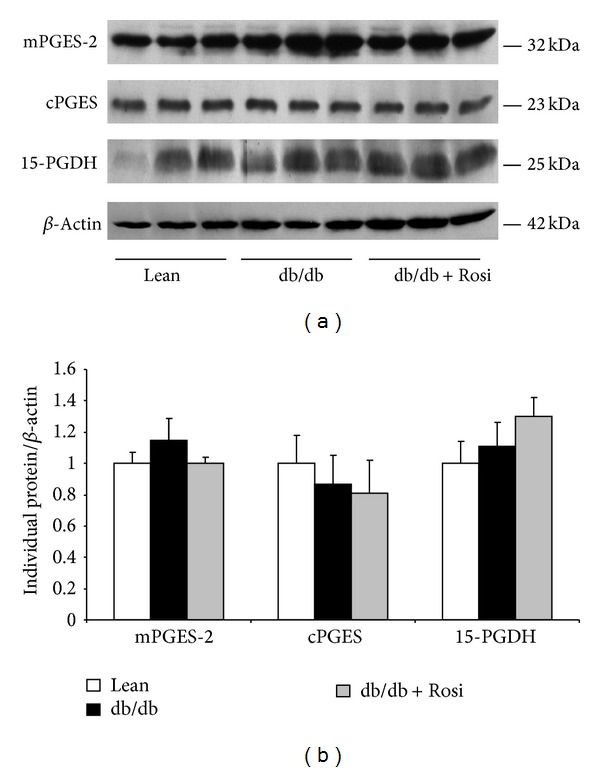
Effect of Rosi treatment on the protein expressions of mPGES-2, cPGES, and 15-PGDH in kidneys of db/db mice. (a) Western blots of mPGES-2, cPGES, 15-PGDH, and *β*-actin. (b) Densitometry of western blots. *N* = 4-5 in each group. Data are mean ± SE.

By Western blotting, the renal mPGES-1 protein was markedly elevated in db/db mice by more than 4-fold, and such an induction was remarkably blunted by Rosi (Figures 2(a) and 2(b)). By immunohistochemistry, mPGES-1 protein was significantly induced in the glomeruli, which was entirely abolished by Rosi treatment (Figures 3(a) and 3(b)). Whereas, the protein expressions of COX-1, mPGES-2, cPGES, and 15-PGDH (Figures 4(a) and 4(b)) were not altered by leptin receptor mutation or Rosi treatment. COX-2 protein expression was significantly higher in the kidney of db/db mice compared with lean mice. However, such an induction of COX-2 protein was not attenuated by Rosi treatment (Figures 2(a) and 2(b)).

### 3.3. Effect of Rosi on the mRNA Expressions of EP1 and EP4 in the Kidneys of db/db Mice

**Figure 5:**
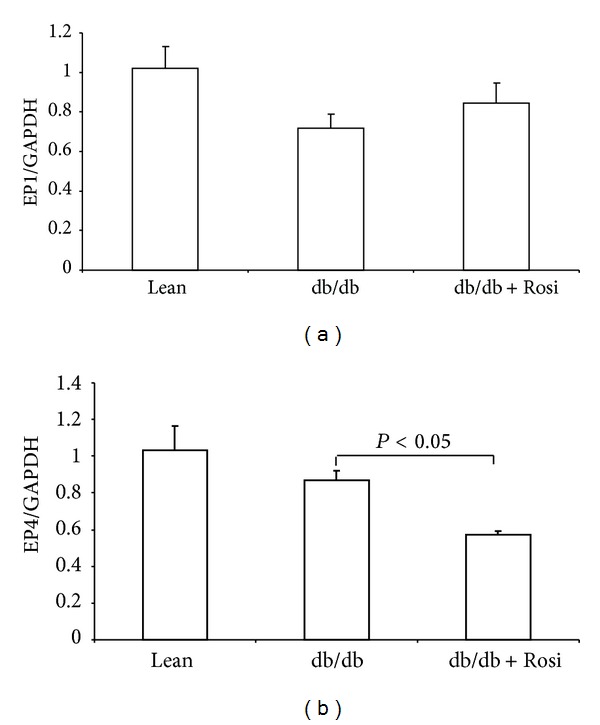
Expressions of EP1 and EP4 mRNA in kidneys of db/db mice following Rosi treatment. (a) qRT-PCR of EP1. (b) qRT-PCR of EP4. *N* = 4-5 in each group. Data are mean ± SE.

Rosi treatment led to significant downregulation of EP4 mRNA expression (Figure 5(b)) in line with the striking reduction of urinary PGE2 and renal mPGES-1 protein. However, EP1 mRNA was not influenced by leptin receptor mutation or Rosi treatment (Figure 5(a)).

### 3.4. Effect of Rosi on the Protein Expression of PPAR*γ* in the Kidneys of db/db Mice

**Figure 6:**
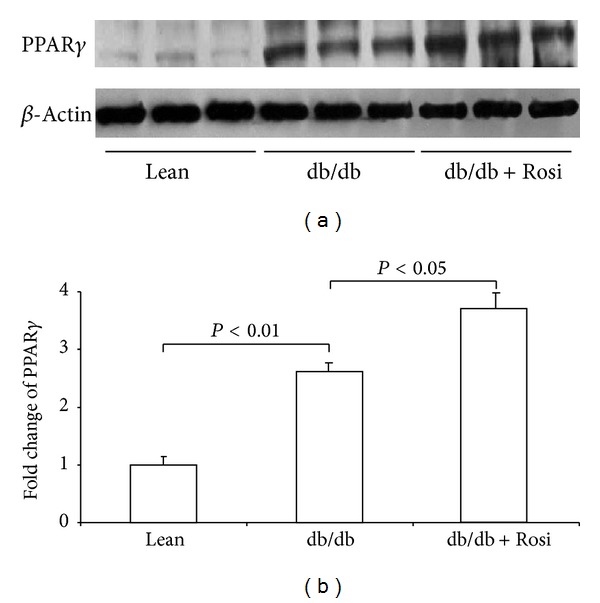
Protein expression of PPAR*γ* in kidneys of db/db mice after Rosi treatment. (a) Western blot of PPAR*γ* and *β*-actin. (b) Densitometry of western blots. *N* = 4-5 for each group. Data are mean ± SE.

By Western blotting, renal PPAR*γ* protein was 2.6-fold higher in db/db mice compared to lean controls and Rosi treatment further increased its expression (Figures 6(a) and 6(b)).

### 3.5. Effect of Rosi Treatment on Glomerular Injury in db/db Mice

**Figure 7:**
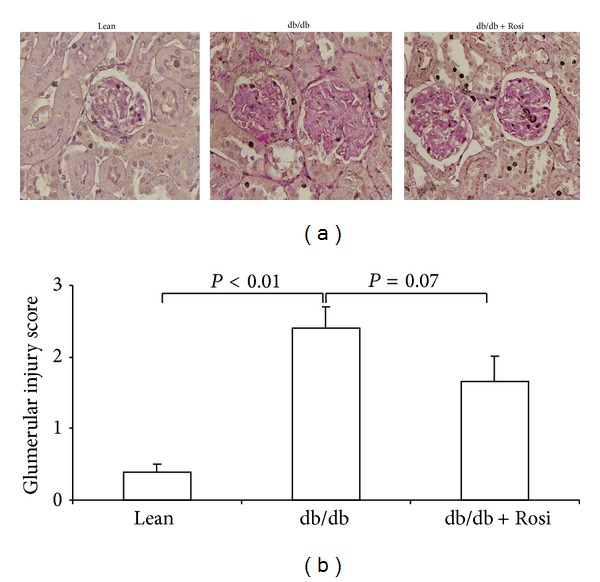
Evaluation of glomerular injury by PAS staining in db/db mice following Rosi treatment. (a) PAS staining. (b) Glomerular injury score. *N*  =  4-5 for each group. Data are mean ± SE.

By PAS staining, we found that the mesangial matrix accumulation was strikingly increased in the db/db mice. Rosi treatment for 1 week resulted in a trend improvement of glomerular injury (*P* = 0.07) (Figures 7(a) and 7(b)).

## 4. Discussion

Type-2 diabetes is in epidemic with rapid increase of obesity worldwide [26]. For patients with type-2 diabetes, DN is a common and severe complication leading to the renal failure and death [19]. Although extensive studies on DN have been performed in past decades, the pathogenic mechanism of this disease is still poorly understood. In the animal studies, leptin receptor mutant db/db mouse is a widely used diabetes model presenting hyperglycemia, hyperlipidemia, obesity, and desensitization of insulin signaling pathway. The kidney injury in db/db mice was well established and the potential pathogenic factors may include the hyperglycemia, lipid disorders, inflammation, and hemodynamic disorders [27, 28].

It has been shown that the renal PGE2 level is elevated in STZ type-1 diabetic animals. Recently, evidence demonstrated a detrimental role of PGE2 in the development of DN in type-1 diabetes via EP1 and/or EP4 receptors [12, 13]. However, whether the renal PGE2 in db/db mice plays a role in diabetic kidney injury is still unknown. Considering the more complicated metabolic disorders in db/db mice, the potential mechanism leading to the kidney injury in this mouse model may significantly differ from STZ-diabetic mice. Therefore, it is worthwhile to examine the regulation of renal PGE2 and its synthetic pathway in db/db mice.

In the present study, we found a remarkable elevation of urinary PGE2 in db/db mice. Meanwhile, the renal mPGES-1 protein was also markedly upregulated in glomeruli, which may contribute to increased renal PGE2 production in these mice. In contrast, renal mPGES-1 expression was not altered in type-1 diabetic mouse model induced by STZ, and the systemic deletion of mPGES-1 played no role in renal PGE2 induction, diabetes onset, and the kidney injury in STZ diabetes (data not shown). This discrepancy of mPGES-1 regulation between two models is possibly due to the different pathogenic mechanism of kidney injury.

Unlike mPGES-1, both mPGES-2 and cPGES were not significantly affected in the kidneys of db/db mice. COX-1 and COX-2 are the upstream enzymes of PGE2 synthases providing the substrate of PGH2 to produce PGE2 and other prostanoids. In agreement with findings from Zucker rats [29], we found that COX-2, but not COX-1, was higher in the kidneys of db/db mice. To further elucidate the potential effect of prostaglandin E2 degradation on the renal PGE2 production, we examined 15-PGDH, an enzyme responsible for the prostaglandins degradation, and found no change in the kidneys of db/db mice.

PPAR*γ* agonists including Rosi and piglitazone are potent agents in treatment of type-2 diabetes mainly via sensitizing the insulin signaling [19, 30–32]. Accumulating evidence demonstrated the beneficial role of PPAR*γ* activation in protecting the diabetic kidney [19–22]. Similarly, our result also showed a trend improvement of glomerular injury following a short period Rosi therapy (7 days). However, the interaction between PPAR*γ* and renal PGE2 production in diabetic kidney is still unidentified. In the present study, we observed that PPAR*γ* agonist Rosi robustly attenuated renal mPGES-1 induction in db/db mice without affecting COX-2 expression. This reduced mPGES-1 protein expression after Rosi treatment could account for the decreased urinary PGE2 excretion. The unaltered high COX-2 expression may contribute to the remained higher urinary PGE2 level in Rosi-treated db/db mice contrasting with the lean controls. The mechanism relating to the Rosi effect on the downregulation of mPGES-1/PGE2 pathway could be complicated. First, this effect might be related to the glycemic control by Rosi. As shown by previous reports, Rosi markedly decreased the blood glucose level in db/db mice [23, 33–35]. Our result also showed that a moderate hyperglycemia in this obese-diabetic db/db mouse strain was almost normalized following 1-week Rosi therapy. Although this antihyperglycemic effect has to be considered for the downregulation of renal mPGES-1/PGE2/pathway, a separate study from our group strongly disagrees with this possibility. In that study, renal mPGES-1 expression was not regulated in STZ-diabetic mouse and mPGES-1 deletion did not affect renal PGE2 induction during 6 weeks of diabetes. Secondly, it is known that COX-2/mPGES-1/PGE2 signaling is highly inducible by various inflammatory stimuli. Moreover, the enhanced inflammatory response in the kidneys of db/db mice was confirmed by numerous studies [16, 36, 37]. Therefore, inflammation does serve as a candidate in stimulating the renal mPGES-1/PGE2 pathway in db/db mice, and the anti-inflammatory property of Rosi might be responsible for the suppression of this pathway. Thirdly, we can not rule out the direct effect of leptin receptor mutation on the renal mPGES-1/PGE2 induction and the potential interaction between leptin receptor and PPAR*γ* in modulation of mPGES-1/PGE2 pathway. At last, the urine flow may also need to be considered. PGE2 exerts diuretic effect leading to the increase of urine flow [38–44]. Oppositely, the urine flow can stimulate renal tubular PGE2 production [45]. However, 1-week Rosi treatment did not affect the urine volume in db/db mice, which largely excluded this possibility.

It had been reported that EP1 and EP4 receptors were involved in the glomerular injury in STZ-diabetic mice [12, 13]. By qRT-PCR, Rosi treatment led to a moderate but significant reduction of EP4 in contrast to the unaltered EP1. In addition, we also examined the urinary excretion of PGD2, 6-keto-PGF1*α*, and TXB2. Unlike PGE2, none of them was influenced by Rosi therapy. These results indicated a selective action of Rosi on renal prostanoids production.

In summary, employing the db/db mice, we found that renal mPGES-1/PGE2 pathway was robustly induced and such an induction was markedly suppressed by PPAR*γ* agonist Rosi. These results highly suggested an interaction between PPAR*γ* and mPGES-1/PGE2/EP4 pathway in the kidneys of db/db mice. The downregulation of mPGES-1/PGE2/EP4 pathway possibly contributes to the protective effect of PPAR*γ* on type-2 diabetes-associated DN. To further validate this hypothesis, blockade of mPGES-1 or EP4 in leptin receptor mutant animals by pharmacological strategies or genetic disruption will be of very importance in the future.
